# Sexual violence and antiretroviral adherence among women of reproductive age in African population‐based surveys: the moderating role of the perinatal phase

**DOI:** 10.1002/jia2.26129

**Published:** 2023-06-12

**Authors:** Leah A. Schrubbe, Heidi Stöckl, Abigail M. Hatcher, Clara Calvert

**Affiliations:** ^1^ Faculty of Epidemiology and Population Health London School of Hygiene & Tropical Medicine (LSHTM) London UK; ^2^ Institute for Medical Information Processing Biometry and Epidemiology, Faculty of Medicine, LMU Munich Munich Germany; ^3^ Pettenkofer School of Public Health Munich Germany; ^4^ Department of Health Behaviour University of North Carolina at Chapel Hill Chapel Hill North Carolina USA; ^5^ Centre for Global Health, Usher Institute University of Edinburgh Edinburgh UK

**Keywords:** HIV, ART adherence, violence against women, sexual violence, pregnancy, breastfeeding

## Abstract

**Introduction:**

Women face challenges in antiretroviral therapy (ART) adherence and achieving viral suppression despite progress in the expansion of HIV treatment. Evidence suggests that violence against women (VAW) is an important determinant of poor ART adherence in women living with HIV (WLH). In our study, we examine the association of sexual VAW and ART adherence among WLH and assess whether this association varies by whether women are pregnant/breastfeeding or not.

**Methods:**

A pooled analysis was conducted among WLH from Population‐Based HIV Impact Assessment cross‐sectional surveys (2015−2018) from nine sub‐Saharan African countries. Logistic regression was used to examine the association between lifetime sexual violence and suboptimal ART adherence (≥1 missed day in the past 30 days) among reproductive age WLH on ART, and to assess whether there was any evidence for interaction by pregnancy/breastfeeding status, after adjusting for key confounders.

**Results:**

A total of 5038 WLH on ART were included. Among all included women, the prevalence of sexual violence was 15.2% (95% confidence interval [CI]: 13.3%−17.1%) and the prevalence of suboptimal ART adherence was 19.8% (95% CI: 18.1%−21.5%). Among only pregnant and breastfeeding women, the prevalence of sexual violence was 13.1% (95% CI: 9.5%−16.8%) and the prevalence of suboptimal ART adherence was 20.1% (95% CI: 15.7%−24.5%). Among all included women, there was evidence for an association between sexual violence and suboptimal ART adherence (adjusted odds ratio [aOR]: 1.69, 95% CI: 1.25−2.28). There was evidence that the association between sexual violence and ART adherence varied by pregnant/breastfeeding status (*p* = 0.004). Pregnant and breastfeeding women with a history of sexual violence had higher odds of suboptimal ART adherence (aOR: 4.11, 95% CI: 2.13−7.92) compared to pregnant and breastfeeding women without a history of sexual violence, while among non‐pregnant and non‐breastfeeding women, this association was attenuated (aOR: 1.39, 95% CI: 1.00−1.93).

**Conclusions:**

Sexual violence is associated with women's suboptimal ART adherence in sub‐Saharan Africa, with a greater effect among pregnant and breastfeeding WLH. To improve women's HIV outcomes and to achieve the elimination of vertical transmission of HIV, violence prevention efforts within maternity services and HIV care and treatment should be a policy priority.

## 1 INTRODUCTION

Women face challenges in antiretroviral therapy (ART) uptake, ART adherence and sustaining viral suppression despite remarkable progress in ART access, including for the prevention of vertical transmission of HIV [1]. While there is scant evidence from population‐based studies examining ART adherence among women of reproductive age in sub‐Saharan Africa, there are more data available specifically focusing on pregnant women [2, 3]. A pooled secondary analysis of population‐based data from 2015 to 2018 from 10 sub‐Saharan African countries, for example, suggests that 79.4% of pregnant and breastfeeding women living with HIV (WLH) had optimal (100%) self‐reported ART adherence [3].

There are a number of barriers that may explain the relatively high levels of suboptimal ART adherence among WLH. A systematic review, which included a majority of studies from sub‐Saharan Africa, found that reported barriers to optimal ART adherence include health service factors, such as distance to clinic and pill burden, as well as socio‐demographic factors, such as young age and low education [4]. Mental health conditions, such as depression, anxiety and substance misuse, are also viewed as important ART correlates [2, 5]. A number of social determinants have received increasing attention in recent literature, including stigma, disclosure, relationship dynamics and male involvement [2, 6, 7, 8, 9, 10].

Violence against women (VAW) is another key social determinant of ART adherence among WLH [11, 12, 13]. A 2015 meta‐analysis found that intimate partner violence was associated with half the odds of self‐reported ART adherence among WLH [11], but the data were drawn from predominately US clinical samples with little evidence around the time of pregnancy. A 2019 scoping review improved representation from African clinical samples and found that WLH who experienced VAW (as defined by the primary articles included in the review, including intimate partner violence or violence regardless of the perpetrator) were less likely to engage in HIV care and treatment, including ART uptake and adherence [14]. Clinical samples have confirmed these associations in African settings [15, 16, 17] but these are methodologically limited as participants are often actively engaged in healthcare.

There are notable recent additions to the population‐based examination of violence and the HIV care and treatment cascade. A pooled population‐based analysis from 30 countries found a significant association between past‐year intimate partner violence and worse viral suppression [12], but did not examine ART adherence specifically. A population‐based study in South Africa among young women found that intimate partner violence reduced viral suppression by 70% [18], but similarly did not include ART adherence measures.

Given the absence of population‐based data of the association between sexual violence regardless of the perpetrator and ART adherence, we aim to examine the association of sexual VAW and suboptimal ART adherence among women of reproductive age through a secondary analysis of the Population‐Based HIV Impact Assessment (PHIA) surveys from nine sub‐Saharan African countries. We additionally assess whether this association is modified by whether women are pregnant/breastfeeding or not.

## 2 METHODS

### 2.1 Survey design

The nationally representative, cross‐sectional PHIA surveys used a stratified multistage sampling design and included household interviews, individual interviews and laboratory testing to measure the HIV epidemic [19]. One woman from each household aged 15 and above was randomly selected to answer interview questions on violence. This analysis included data from Cameroon, Cote D'Ivoire, Eswatini, Lesotho, Malawi, Namibia, Uganda, Zambia and Zimbabwe, which were collected between 2015 and 2018 (further country‐level details in Table S1).

Women were interviewed by trained survey staff using tablets on socio‐demographics, relationship dynamics, and HIV care and treatment. Blood samples were tested for HIV serostatus, recency of HIV infection, HIV RNA viral load and selected antiretroviral (ARV) drug presence. HIV‐1 serostatus testing was conducted using country algorithms that generally included a combination of home‐based rapid HIV testing and confirmatory laboratory‐based testing. The recency of HIV infection was determined by a combination algorithm of Limiting Antigen Enzyme Immunoassay (LAg‐Avidity EIA), viral load and ARV results. The LAg‐Avidity EIA threshold corresponded to a mean duration since seroconversion between 130 and 153 days depending on the HIV subtype. Further details of PHIA methods can be found in final reports available online [20].

### 2.2 Study population

This secondary analysis was restricted to HIV‐positive women of reproductive age (15–49) on ART with available violence and ART adherence data from the PHIA surveys. HIV‐positive serostatus was based on laboratory testing results. ART status was based on a combination of self‐report and ARV detection through blood testing. ARV detection was conducted on dried blood spot specimens using high‐resolution liquid chromatography with tandem mass spectrometry. This highly specific and sensitive assay was set with a limit of detection of 0.02 μg/ml for each drug.

### 2.3 Exposure of interest

We categorized lifetime sexual violence as “yes” if a participant self‐reported ever having experienced sexual violence or “no” if they reported never having experienced any sexual violence. Questions asked about sexual violence included being touched without permission, being pressured into sex, attempted forced sex and physically forced sex. Violence question wording, answer choices and types of violence included varied among countries. Three of nine countries only included two of the four sexual violence measures. Table S2 presents sexual violence measures available in each country. In this sample, 90 women (1.6%) refused the violence module questions.

In a sensitivity analysis, we repeated our analyses using a definition of sexual violence only including being pressured into sex, attempted forced sex and physically forced sex.

### 2.4 Outcome of interest

Self‐reported optimal ART adherence was defined as no missed days in the past 30 days [8, 21, 22], whereas suboptimal ART adherence was defined as any (≥1) missed days in the past 30 days. Of the women of reproductive age living with HIV on ART and with available violence data, 385 women (7.1%) were missing ART adherence data. Of these, 293 did not self‐report their HIV‐positive status on the survey (stated HIV negative or don't know/refused) and thus were not asked the question, but had ARVs detected from the blood test.

In a sensitivity analysis, we repeated our analyses using a more relaxed definition of ART adherence of ≥2 missed days for suboptimal ART adherence. There is evidence that perfect adherence may not be required for successful virologic outcomes [23], and a more relaxed definition may be considered [24, 25].

### 2.5 Socio‐demographic confounders

Socio‐demographic characteristics were considered as potential confounders of the association between sexual violence and suboptimal ART adherence. Data were available on age, education, employment, wealth, marital status, urban/rural residence and region. Employment was defined as paid work in the last 12 months for which a paycheck, cash or goods was received as payment. An asset‐based wealth index was categorized into wealth index scores and then into quintiles [26].

Due to small numbers in some countries (Table S1), we could not include country as a confounder for analyses using data pooled across all countries. We, therefore, created a variable for region based on the United Nations subregions for Africa [27] with Western and Central Africa combined into one category due to data sparsity (Table S1).

### 2.6 Other measures

Women self‐reported current pregnancy status and breastfeeding status, and we used this in the analysis to look at the association between sexual violence and ART adherence by whether women were pregnant/breastfeeding or not pregnant/breastfeeding. Women reporting as not pregnant or not breastfeeding but missing data on the other status were coded as not pregnant/breastfeeding.

HIV‐ and relationship‐related data used for descriptive analysis were available on healthcare power, financial power, partner disclosure, recency of HIV infection, ART duration and viral load suppression. Healthcare power and financial power were based on interview responses on who usually made decisions about their healthcare and decided how the money they received was spent; these questions were only asked of women whose marital status was currently married or living together. Partner disclosure included disclosure to a spouse or sex partner. ART duration was based on self‐reported ART start date. ART duration was not considered as a confounder since violence has been associated with ART initiation [14] and could be on the causal pathway. Based on WHO guidelines, viral load suppression was defined as <1000 copies/ml and unsuppressed viral load as ≥1000 copies/ml [28].

### 2.7 Statistical analysis

All statistical analyses were done using R version 4.0.2 (2020, R Foundation for Statistical Computing, Vienna, Austria) using data pooled across all the countries unless specified otherwise. The complex multistage cluster sample design was accounted for with sampling weights for the violence module. Weighting accounted for selection probability and non‐response. Jackknife variance estimation was used for this pooled multi‐country cross‐sectional secondary analysis. Provided PHIA weights were denormalized accounting for differential country population size [29].

Missing data were explored for patterns of non‐response and, in the absence of any obvious patterns of missingness, a complete case analysis was conducted. In descriptive analyses, the unweighted number of participants and weighted proportions were calculated for the key socio‐demographic, HIV‐related and relationship‐related characteristics, for all women of reproductive age and stratified by whether women were pregnant/breastfeeding or not. The weighted prevalence of sexual violence and ART adherence were calculated, overall, by region and by country, and by whether women were pregnant/breastfeeding or not.

We used logistic regression to obtain crude associations between key socio‐demographic characteristics and ART adherence. Using multivariable logistic regression, a model examining the association between sexual violence and ART adherence was fitted with sexual violence alone, then a priori confounders (age, region and education) were added. Then, other potential socio‐demographic confounders were added to the model one at a time starting with those with the smallest *p*‐value in crude analyses. The confounder was retained in the model if the odds ratio (OR) changed by >10%. An interaction term was subsequently included in the multivariable logistic regression model to examine whether the association between sexual violence and ART adherence varied by whether women were currently pregnant/breastfeeding or not. Women missing both pregnancy and breastfeeding status were excluded from this interaction analysis. We presented adjusted odds ratios (aORs), 95% confidence interval (CI) and Wald test *p*‐values.

### 2.8 Ethical approval

Local institutional review boards (IRB) and the IRB at the Centers for Disease Control and Prevention, Columbia University Medical Center, and Westat approved the PHIA surveys and informed consent was obtained from participants. The Research Ethics Committee at the London School of Hygiene and Tropical Medicine provided ethical approval for this secondary analysis.

## 3 RESULTS

### 3.1 Characteristics of study population

A total of 5038 WLH and on ART were included (Table 1) from the 13,709 total WLH sampled in the PHIA surveys. Their mean age was 35, roughly half (51.7%) had no education or only primary education, and approximately half (55.6%) were of rural residence. Six were missing pregnancy/breastfeeding status, 82.2% (*n* = 4233) were not pregnant or breastfeeding, and 17.8% (*n* = 799) were pregnant or breastfeeding (232 pregnant, 566 breastfeeding and 1 both pregnant and breastfeeding). Of all women, 99.9% (*n* = 5033) had long‐term infections, and 66.7% (*n* = 3281) had been on ART for ≥24 months. Participant characteristics broken down by ART adherence status are presented in Table S3.

**Table 1:** Sample characteristics among women of reproductive age living with HIV and on ART, by pregnancy/breastfeeding status^a^

	All (*n* = 5038) no. (%)^b^	Not pregnant or breastfeeding (*n* = 4233) no. (%)^b^	Pregnant or breastfeeding (*n* = 799) no. (%)^b^
Socio‐demographic factors			
Age (years)			
15−19	95 (3.1%)	78 (2.9%)	17 (3.8%)
20−24	356 (9.6%)	228 (7.4%)	127 (19.9%)
25−29	754 (13.8%)	543 (11.6%)	210 (23.6%)
30−34	1062 (18.6%)	838 (17.2%)	223 (24.9%)
35−39	1154 (22.5%)	990 (23.2%)	163 (19.7%)
40−49	1617 (32.4%)	1556 (37.7%)	59 (8.0%)
Highest level of education attended			
None	303 (8.1%)	250 (8.0%)	53 (8.7%)
Primary education	2161 (43.6%)	1784 (42.2%)	373 (49.8%)
Secondary education	2146 (41.9%)	1811 (42.7%)	333 (38.1%)
Post‐secondary education	424 (6.4%)	385 (7.1%)	39 (3.4%)
Missing	4	3	1
Paid work in the last 12 months			
Yes	1765 (35.6%)	1555 (3.0%)	209 (29.8%)
No	3270 (65.4%)	2675 (63.0%)	590 (70.2%)
Missing	3	3	0
Wealth quintile			
Lowest	1084 (16.9%)	865 (15.7%)	217 (22.7%)
Second	978 (18.7%)	823 (18.6%)	155 (19.5%)
Middle	1023 (19.9%)	845 (19.3%)	177 (23.1%)
Fourth	990 (21.1%)	844 (20.7%)	143 (22.6%)
Highest	958 (23.3%)	852 (25.8%)	106 (12.1%)
Missing	5	4	1
Marital status			
Married/living together	2871 (55.5%)	2266 (51.0%)	600 (76.1%)
Divorced/separated/widowed	1232 (30.9%)	1139 (34.3%)	92 (15.4%)
Never married/lived together	918 (13.6%)	814 (14.7%)	104 (8.4%)
Missing	17	14	3
Urban/rural residence			
Urban	2094 (44.4%)	1796 (46.6%)	295 (34.1%)
Rural	2944 (55.6%)	2437 (53.4%)	504 (65.9%)
Region			
Southern Africa	2566 (17.7%)	2264 (19.3%)	301 (10.7%)
Western/Central Africa	172 (10.9%)	151 (11.7%)	21 (7.1%)
Eastern Africa	2300 (71.4%)	1818 (69.0%)	477 (82.2%)
Relationship‐related factors			
Healthcare power^c^			
Self	988 (31.4%)	807 (31.8%)	180 (30.2%)
Spouse/partner or someone else	442 (22.2%)	330 (21.7%)	111 (23.7%)
Both self and spouse/partner	1438 (46.4%)	1127 (46.4%)	308 (46.2%)
N/A, not currently married/living together	2150	1953	196
Missing	20	16	4
Financial power^c^			
Self	594 (21.2%)	495 (20.9%)	97 (22.0%)
Spouse/partner or someone else	546 (23.7%)	400 (22.9%)	145 (26.5%)
Both self and spouse/partner	1723 (55.0%)	1364 (56.2%)	357 (51.5%)
N/A, not currently married/living together or doesn't receive money	2154	1957	196
Missing	21	17	4
Disclosed HIV‐positive status to partner			
Yes	2721 (50.7%)	2197 (47.7%)	519 (64.5%)
No	2315 (49.3%)	2034 (52.3%)	280 (35.5%)
Missing	2	2	0
Lifetime sexual violence			
Yes	605 (15.2%)	502 (15.6%)	102 (13.1%)
No	4433 (84.8%)	3731 (84.4%)	697 (86.9%)
HIV‐related factors			
ART duration			
<12 months	909 (19.8%)	694 (18.5%)	213 (25.7%)
12−23 months	649 (13.5%)	490 (12.0%)	158 (20.3%)
≥24 months	3281 (66.7%)	2887 (69.5%)	391 (54.0%)
Missing	199	162	37
Viral suppression			
Suppressed	4466 (86.4%)	3764 (86.3%)	696 (86.9%)
Not suppressed	572 (13.6%)	469 (13.7%)	103 (13.1%)

Abbreviations: ART, antiretroviral therapy; HIV, human immunodeficiency virus.

^a^ Unweighted number of participants and weighted percentages and *p*‐values are reported. Percentages might not total 100% due to rounding. *n* = 6 missing pregnancy/breastfeeding status.

^b^ Missing values and not applicable values not included in %.

^c^ Question only asked of those with marital status of currently married or living together.

Among pregnant/breastfeeding women, compared to non‐pregnant and non‐breastfeeding women, a higher proportion were unemployed (70.2% vs. 63.0%), married/living together (76.1% vs. 51.0%) and had disclosed their HIV‐positive status to their partner (64.5% vs. 47.7%).

**Figure 1:**
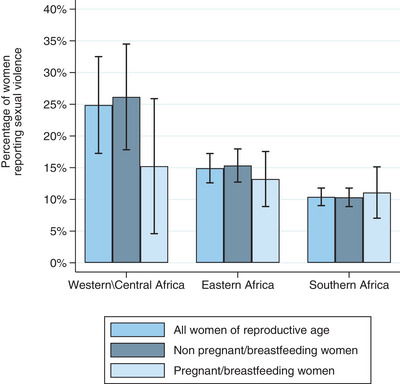
Sexual violence prevalence by region and by pregnant/breastfeeding status.

Among all included women, the prevalence of sexual violence was 15.2% (95% CI: 13.3%−17.1%). This differed by region (*p*<0.001), ranging from 10.4% in Southern Africa to 24.9% in Western/Central Africa (Figure 1). Among only currently pregnant and breastfeeding women, the prevalence of sexual violence was 13.1% (95% CI: 9.5%−16.8%) with no evidence this varied by region (*p* = 0.66). Among non‐pregnant and non‐breastfeeding women, the prevalence of sexual violence was 15.6% (95% CI: 13.6%−17.7%). This differed by region (*p*<0.001), ranging from 10.3% in Southern Africa to 26.2% in Western/Central Africa. Country‐level estimates are provided in Table S4.

**Figure 2:**
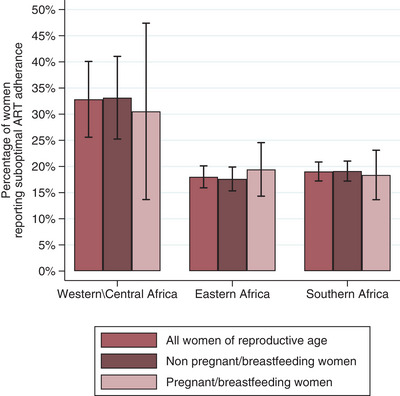
Suboptimal ART adherence prevalence by region and by pregnant/breastfeeding status.

Among all included women, the prevalence of suboptimal ART adherence was 19.8% (95% CI: 18.1%−21.5%). This differed by region (*p*<0.001), ranging from 18.0% in Eastern Africa to 32.8% in Western Africa (Figure 2). Among only currently pregnant and breastfeeding women, the prevalence of suboptimal ART adherence was 20.1% (95% CI: 15.7%−24.5%) with no evidence this varied by region (*p* = 0.37). Among non‐pregnant and non‐breastfeeding women, the prevalence of suboptimal ART adherence was 19.7% (95% CI: 17.9%−21.6%). This differed by region (*p* = 0.001), ranging from 17.6% in Southern Africa to 33.1% in Western/Central Africa. Country‐level estimates are provided in Table S4.

### 3.2 Association between lifetime sexual violence and ART adherence in women of reproductive age living with HIV and on ART

In crude analyses, there was evidence (*p*<0.001) for a crude association between sexual violence and ART adherence with women who reported a lifetime history of sexual violence having almost two times (OR: 1.81, 95% CI: 1.33−2.48) the odds of reported suboptimal ART adherence compared to women who did not report a history of sexual violence (Table 2).

**Table 2:** Adjusted association of sexual violence with suboptimal ART adherence among women of reproductive age living with HIV and on ART^a^

	Number with suboptimal ART adherence/*N* (%)	Crude OR (95% CI)	*p*‐value^b^	aOR (95% CI)^c^	*p*‐value^b^
Lifetime sexual violence					
No	761/4433 (18.2%)	1	<0.001	1	0.001
Yes	178/605 (28.8%)	1.81 (1.33−2.48)		1.69 (1.25−2.28)	

Abbreviations: aOR, adjusted odds ratio; ART, antiretroviral therapy; CI, confidence interval; HIV, human immunodeficiency virus; OR, odds ratio.

^a^ Outcome was ≥1 missed days of ART in the past 30 days. Final adjusted model included *n* = 5034 participants.

^b^ Wald test *p*‐value.

^c^ Model adjusted for age, region and education.

In multivariable analyses, after adjusting for age, region and education, the association between sexual violence and ART adherence remained (aOR: 1.69, 95% CI: 1.25−2.28, *p* = 0.001) (Table 2). Other socio‐demographic factors explored were not found to be confounders (Table S5).

### 3.3 Association between lifetime sexual violence and ART adherence in pregnant and breastfeeding WLH and on ART

Among women with a history of sexual violence, a higher proportion of pregnant and breastfeeding women had suboptimal ART adherence compared to non‐pregnant and non‐breastfeeding women (43.7% vs. 26.1%). There was evidence for effect modification of the association between sexual violence and ART adherence by pregnant/breastfeeding status (*p* = 0.004) (Table 3). After controlling for age, region and education, pregnant and breastfeeding women with a history of sexual violence had four times the odds (aOR: 4.11, 95% CI: 2.13−7.92) of suboptimal ART adherence compared to pregnant and breastfeeding women without a history of sexual violence. Among non‐pregnant and non‐breastfeeding women, the adjusted OR was lower (aOR: 1.39, 95% CI: 1.00−1.93). Models adjusting for socio‐demographic characteristics not considered confounders in this study are provided in Table S6.

**Table 3:** Association between sexual violence and ART adherence by pregnant/breastfeeding status among women living with HIV and on ART, by whether women were pregnant/breastfeeding or not pregnant/breastfeeding^a^

	Number with optimal ART adherence/*N* (%)	Number with suboptimal ART adherence/*N* (%)	aOR (95% CI)^b^	*p*‐value^c^
Interaction by pregnancy/breastfeeding status				0.004
Pregnant/breastfeeding				
No sexual violence	580 (83.4%)	117 (16.6%)	1	
Yes, sexual violence	69 (56.3%)	33 (43.7%)	4.11 (2.13−7.92)	<0.001
Not pregnant/breastfeeding				
No sexual violence	3089 (81.5%)	642 (18.5%)	1	
Yes, sexual violence	357 (73.9%)	145 (26.1%)	1.39 (1.00−1.93)	0.05

Abbreviations: aOR, adjusted odds ratio; ART, antiretroviral therapy; CI, confidence interval; HIV, human immunodeficiency virus.

^a^ Outcome was ≥1 missed days of ART in the past 30 days. Final adjusted model included *n* = 5028 participants.

^b^ Model adjusted for age, region and education.

^c^ Wald test *p*‐value.

### 3.4 Sensitivity analyses

The association between sexual violence and suboptimal ART adherence remained, even after using a definition of pressured, attempted forced, or physically forced sex for sexual violence; however, the crude and adjusted ORs were slightly lower than in the main model (Table S7). Using a definition of ≥2 missed days for suboptimal ART adherence, the crude and adjusted ORs were slightly higher than in the main model (Table S8).

## 4 DISCUSSION

Using population‐based data pooled from nine sub‐Saharan African countries, we found strong evidence that sexual violence is associated with suboptimal ART adherence after controlling for confounding factors. Women who experienced sexual violence had nearly twice the odds of reporting suboptimal ART adherence, but this increased to four times the odds of suboptimal ART adherence when restricting to pregnant and breastfeeding women. These findings suggest that sexual VAW has a detrimental impact on ART adherence, particularly among pregnant and breastfeeding women.

Our findings build on existing quantitative evidence from sub‐Saharan Africa that also established that VAW has a negative impact on ART adherence for WLH. Multiple studies have found that WLH who have experienced intimate partner violence, or VAW more broadly, are more likely to have challenges with ART adherence and viral suppression [11, 12, 14, 16]. Though the evidence on pathways linking VAW and ART adherence in pregnant and breastfeeding women is limited, a few qualitative studies from Johannesburg, South Africa (*n* = 32 and *n* = 38) and Lusaka, Zambia (*n* = 32) found that intimate partner violence worsens HIV‐related health in pregnant and postpartum women through leading to women's increased poor mental health, such as depression and anxiety [30, 31, 32]. There is emerging literature to support a finding that lifetime exposure to violence is related to ART adherence. A recent quantitative study from Johannesburg, South Africa found that past‐year intimate partner violence had a direct and indirect effect on perinatal ART adherence through perinatal depression, anxiety and post‐traumatic stress disorder symptoms [16]. Outside of violence exposure, physiologic changes from the pregnancy itself may contribute to common mental disorders or may exacerbate pre‐existing symptoms [33, 34, 35]. Lifetime exposure to traumatic events, including intimate partner violence and/or childhood abuse, may predispose women to perinatal mental disorders [33, 34, 35]. Other potential mechanisms between violence exposure and ART adherence identified among women, including pregnant and postpartum women, include partner non‐disclosure [14, 31], partner control [31], alcohol use [2, 36] and poor antenatal care attendance [2]. In particular, non‐disclosure can lead to challenges in women's HIV care due to fear of partner's reaction, including violence, and make it harder for women to adhere to medication due to having to hide it [14, 31]. Longitudinal studies in sub‐Saharan Africa are needed to determine modifiable mechanisms through which VAW impacts negatively on HIV care and treatment with attention to how these vary between pregnant or breastfeeding women and non‐pregnant and non‐breastfeeding women. In addition, future research should aim to identify how partner‐enacted violence may differ from non‐partner violence in terms of women's mental health and subsequent HIV outcomes.

Our finding that pregnant and breastfeeding women with a history of sexual violence had four times the odds of suboptimal ART adherence provides further evidence for the need for intervention during maternity care. To improve women's HIV outcomes, eliminate vertical transmission of HIV and reduce other potential transmission of HIV during the pregnancy/breastfeeding period, a focus on addressing violence, mental health and HIV during antenatal and postnatal care is crucial. Through repeated interactions with the healthcare system during this period, antenatal care presents a window of opportunity for healthcare workers to jointly address violence and HIV for pregnant WLH [31]. A nurse‐led safety planning intervention in South Africa, for example, reduced perinatal exposure to intimate partner violence [37].

Creating safe spaces for women to disclose their experiences with violence to healthcare providers could help identify women at risk of poor engagement in HIV care and provide them with suitable interventions. Addressing mental health through trauma‐informed programmes may help to disentangle a potential pathway through which violence impacts HIV treatment [31, 37]. Additionally, the expansion of gender equality programmes as well as violence prevention efforts has the potential to reduce violence and improve HIV outcomes. Community‐based interventions, such as “SASA!” and “SHARE,” have been shown to reduce intimate partner violence and HIV incidence [38, 39]. Through community mobilization, these interventions address inequitable gender dynamics, promote equitable social norms and behaviours, and combine these with HIV‐related strategies to help address violence and reduce HIV vulnerability for women. In addition, the IMAGE intervention, which uses microfinance alongside HIV and gender training, has been shown to reduce past‐year intimate partner violence and HIV risk behaviours in young women [40, 41]. Future research could take lessons learned from these interventions to apply to VAW and HIV care and treatment outcomes for WLH.

There were strengths and limitations to this analysis. The population‐based design of PHIA surveys and large sample size are strengths. However, self‐reported data included such as sexual violence and ART adherence could have been subject to social desirability bias. Overall, 7.1% of HIV‐positive women of reproductive age with violence data were missing ART adherence data and were excluded from this analysis. It is possible that these women were more likely to have suboptimal ART adherence leading to underestimates of suboptimal ART adherence. However, the levels of missing data were similar for both women who reported lifetime sexual violence and women who did not report lifetime sexual violence so this will not explain the association we observe between sexual violence and suboptimal ART adherence. Underreporting of sexual violence due to stigma, fear of reporting and social/cultural normalization of violence is common and this could have affected the data [42]. In addition, the violence module's question wording, answer choices and types of violence included varied among countries. Three of nine countries did not include all four sexual violence measures. As not all sexual violence measures were included in all countries, violence prevalence was very likely underestimated, and this may have affected the results. PHIA surveys only measured intimate partner violence in the past 12 months, in select countries, and women's relationship to perpetrators was collected sporadically, so we were unable to break down lifetime partner versus non‐partner sexual violence. Physical violence was not available in every country and thus was not included. Psychological violence was only measured in one country and thus not included. While pooling data from multiple countries has benefits for applicability across countries and settings, increasing sample size and producing regional estimates, there are limitations to interpretation. Considerable cultural, political and policy differences exist among countries. These differences are likely to drive some of the different patterns we observe across regions in this analysis, for example, in sexual violence and ART adherence prevalence. The cross‐sectional nature of the data limits the interpretation of causality and reverse causality remains a possibility. It is plausible that women with suboptimal ART adherence were more likely to subsequently experience sexual violence. However, the ART adherence outcome referring to the past 30 days and the sexual violence exposure referring to lifetime risk reduce the risk of reverse causality. We cannot rule out residual confounding for the association between sexual violence and ART adherence. In particular, travel time to the clinic would have been useful to examine, but these data were only available in a few countries. Finally, data were only available up to 2018 at the time of this study, meaning we could not explore the impact of the COVID‐19 pandemic on levels of sexual violence, ART adherence and the association between these. The UN has reported that VAW intensified during the pandemic [43] and studies have indicated that ART adherence declined at least in some settings [44, 45]. Future studies need to explore the longer‐term impacts of the COVID‐19 pandemic on the association between sexual violence and ART adherence.

## 5 CONCLUSIONS

We found strong evidence for an association between lifetime sexual violence and suboptimal ART adherence. The effect is particularly substantial among the subgroup of pregnant and breastfeeding WLH. To improve women's HIV outcomes and to reach the elimination of vertical transmission of HIV, implementation and/or expansion of gender equality programmes as well as violence prevention efforts should be considered. Addressing mental health and violence in antenatal care and HIV care may help to improve HIV care and treatment outcomes for women at risk. Ending VAW is important on its own from a human rights perspective but will also have implications for helping to end the HIV epidemic and improve health for women and children worldwide.

## AUTHORS’ CONTRIBUTIONS

LAS, HS, AMH and CC conceptualized the analysis and study design. LAS conducted the analysis and drafted the original manuscript draft. All authors assisted with data interpretation and manuscript editing. All authors read and approved the final article.

## COMPETING INTERESTS

There are no competing interests.

## Supporting information

Supporting Information

## Data Availability

The data that support the findings of this study are available upon request from the PHIA project at https://phia‐data.icap.columbia.edu/.
