# Supplementary material for: Sexual violence and antiretroviral adherence among women of reproductive age in African population‐based surveys: the moderating role of the perinatal phase
Source: J Int AIDS Soc. 2023 Jun 12;26(6):e26129. doi: 10.1002/jia2.26129 (PMC10258861; doi:10.1002/jia2.26129)
Supplement: Supplementary file 1 — Supporting Information [file JIA2-26-e26129-s001.docx]

**SUPPORTING INFORMATION**

**Table S1. Summary of number of included women, by country, and by pregnant/breastfeeding status^†^**

| **Country and years of data collection** | **Region** | **All**  **(n=5038)**  **no. (%)** | **Number not pregnant or breastfeeding (n=4233)**  **no. (%)** | **Number pregnant or breastfeeding**  **(n=799)**  **no. (%)** |
| --- | --- | --- | --- | --- |
| **Cameroon**  **(2017-2018)** | Western and Central Africa | 126 (7.3%) | 113 (8.0%) | 13 (4.0%) |
| **Cote D’Ivoire**  **(2017-2018)** | Western and Central Africa | 46 (3.6%) | 38 (3.7%) | 8 (3.2%) |
| **Eswatini**  **(2016-2017)** | Southern Africa | 786 (5.5%) | 692 (6.0%) | 94 (3.5%) |
| **Lesotho**  **(2016-2017)** | Southern Africa | 1031 (7.6%) | 920 (8.4%) | 111 (4.1%) |
| **Malawi**  **(2015-2016)** | Eastern Africa | 733 (21.1%) | 556 (19.4%) | 176 (28.7%) |
| **Namibia**  **(2017)** | Southern Africa | 749 (4.6%) | 652 (4.9%) | 96 (3.0%) |
| **Uganda**  **(2016-2017)** | Eastern Africa | 29 (4.3%) | 18 (3.7%) | 11 (7.1%) |
| **Zambia**  **(2016)** | Eastern Africa | 626 (20.2%) | 497 (20.0%) | 128 (21.5%) |
| **Zimbabwe**  **(2015-2016)** | Eastern Africa | 912 (25.8%) | 747 (26.0%) | 162 (24.8%) |

^†^Unweighted number of participants and weighted percentages reported. Percentages might not total 100% due to rounding. n=6 missing pregnancy/breastfeeding status.

**Table S2. Sexual violence measures available by country^†^**

| **Country** | **Touch without permission** | **Attempted forced sex through harassment, threats, tricks, or physical force** | **Physically forced sex** | **Pressured sex through harassment, threats, or tricks** |
| --- | --- | --- | --- | --- |
| **Cameroon** | Yes | Yes | Yes | Yes |
| **Cote D’Ivoire** | Yes | Yes | Yes | Yes |
| **Eswatini** | Yes | No | Yes | No |
| **Lesotho** | No | No | Yes | Yes |
| **Malawi** | Yes | Yes | Yes | Yes |
| **Namibia** | No | No | Yes | Yes |
| **Uganda** | Yes | Yes | Yes | Yes |
| **Zambia** | Yes | Yes | Yes | Yes |
| **Zimbabwe** | Yes | Yes | Yes | Yes |

^†^some question wording varied slightly among countries

**Table S3. Sample characteristics by ART adherence among women of reproductive age living with HIV and on ART^†^**

|  | **All**  **(n=5038)**  **no. (%)^‡^** | **Number with optimal ART adherence (n=4099)**  **no. (%)^‡^** | **Number with suboptimal ART adherence (n=939)**  **no. (%)^‡^** |
| --- | --- | --- | --- |
| **Sociodemographic Factors** |  |  |  |
| **Age (years)** |  |  |  |
| 15-19 | 95 (3.1%) | 73 (70.5%) | 22 (29.5%) |
| 20-24 | 356 (9.6%) | 258 (75.4%) | 98 (24.6%) |
| 25-29 | 754 (13.8%) | 599 (77.7%) | 155 (22.3%) |
| 30-34 | 1062 (18.6%) | 871 (80.1%) | 191 (19.9%) |
| 35-39 | 1154 (22.5%) | 949 (80.9%) | 205 (19.1%) |
| 40-49 | 1617 (32.4%) | 1349 (83.2%) | 268 (16.8%) |
| **Highest level of education attended** |  |  |  |
| None | 303 (8.1%) | 244 (77.2%) | 59 (22.8%) |
| Primary education | 2161 (43.6%) | 1731 (76.5%) | 430 (23.5%) |
| Secondary education | 2146 (41.9%) | 1789 (85.2%) | 357 (14.8%) |
| Post-secondary education | 424 (6.4%) | 331 (76.4%) | 93 (23.6%) |
| Missing | 4 | 4 | 0 |
| **Paid work in last 12 months** |  |  |  |
| Yes | 1765 (35.6%) | 1414 (77.5%) | 351 (22.5%) |
| No | 3270 (65.4%) | 2682 (81.6%) | 588 (18.4%) |
| Missing | 3 | 3 | 0 |
| **Wealth quintile** |  |  |  |
| Lowest | 1084 (16.9%) | 903 (82.0%) | 181 (18.0%) |
| Second | 978 (18.7%) | 802 (82.1%) | 176 (17.9%) |
| Middle | 1023 (19.9%) | 841 (79.1%) | 182 (20.9%) |
| Fourth | 990 (21.1%) | 801 (80.4%) | 189 (19.6%) |
| Highest | 958 (23.3%) | 747 (78.1%) | 211 (21.9%) |
| Missing | 5 | 5 | 0 |
| **Marital status** |  |  |  |
| Married/Living together | 2871 (55.5%) | 2347 (80.1%) | 524 (19.9%) |
| Divorced/Separated/ Widowed | 1232 (30.9%) | 988 (80.3%) | 244 (19.7%) |
| Never married/lived together | 918 (13.6%) | 750 (80.5%) | 168 (19.5%) |
| Missing | 17 | 14 | 3 |
| **Urban/Rural Residence** |  |  |  |
| Urban | 2094 (44.4%) | 1661 (79.6%) | 433 (20.4%) |
| Rural | 2944 (55.6%) | 2438 (80.7%) | 506 (19.3%) |
| **Region** |  |  |  |
| Southern Africa | 2566 (17.7%) | 2087 (81.0%) | 479 (19.0%) |
| Western/Central Africa | 172 (10.9%) | 109 (67.2%) | 63 (32.8%) |
| Eastern Africa | 2300 (71.4%) | 1903 (82.0%) | 397 (18.0%) |
| **Relationship-related Factors** |  |  |  |
| **Healthcare power^§^** |  |  |  |
| Self | 988 (31.4%) | 802 (80.1%) | 186 (19.9%) |
| Spouse/partner or someone else | 442 (22.2%) | 356 (76.3%) | 86 (23.7%) |
| Both self and spouse/partner | 1438 (46.4%) | 1187 (81.8%) | 251 (18.2%) |
| N/A, not currently married/living together | 2150 | 1738 | 412 |
| Missing | 20 | 16 | 4 |
| **Financial power^§^** |  |  |  |
| Self | 594 (21.2%) | 482 (77.2%) | 112 (22.8%) |
| Spouse/partner or someone else | 546 (23.7%) | 439 (78.5%) | 107 (21.5%) |
| Both self and spouse/partner | 1723 (55.0%) | 1418 (81.8%) | 305 (18.2%) |
| N/A, not currently married/living together or doesn’t receive money | 2154 | 1742 | 412 |
| Missing | 21 | 18 | 3 |
| **Disclosed HIV+ status to partner** |  |  |  |
| Yes | 2721 (50.7%) | 2229 (80.9%) | 492 (19.1%) |
| No | 2315 (49.3%) | 1868 (79.4%) | 447 (20.6%) |
| Missing | 2 | 2 | 0 |
| **Lifetime sexual violence** |  |  |  |
| Yes | 605 (15.2%) | 427 (71.2%) | 178 (28.8%) |
| No | 4433 (84.8%) | 3672 (81.8%) | 761 (18.2%) |
| **HIV-related Factors** |  |  |  |
| **ART duration** |  |  |  |
| <12 months | 909 (19.8%) | 752 (82.6%) | 157 (17.4%) |
| 12-23 months | 649 (13.5%) | 531 (80.0%) | 118 (20.0%) |
| ≥24 months | 3281 (66.7%) | 2656 (79.8%) | 625 (20.2%) |
| Missing | 199 | 160 | 39 |

ART=antiretroviral therapy. HIV=human immunodeficiency virus.

^†^Suboptimal ART adherence was ≥1 missed days of ART in the past 30 days.

^‡^Unweighted number of participants and weighted percentages and p-values are reported. Percentages might not total 100% due to rounding. Missing values and not applicable values not included in %.

^§^Question only asked of those with marital status of currently married or living together

**Table S4. Sexual violence and ART adherence prevalence by country and by pregnant/breastfeeding status**

| **Country and group** | **Number of women with lifetime sexual violence** | **Sexual violence prevalence (95% CI)** | **Number of women with suboptimal ART adherence** | **Suboptimal ART adherence prevalence (95% CI)** |
| --- | --- | --- | --- | --- |
| **Cameroon** |  |  |  |  |
| Non-pregnant/breastfeeding women | 26 | 27.6% (15.5%-39.8%) | 41 | 31.4% (20.0%-42.9%) |
| Pregnant/breastfeeding women | 5 | 22.3% (1.1%-43.4%) | 5 | 30.2% (0%-62.0%) |
| Women of reproductive age | 31 | 27.1% (15.8%-38.5%) | 46 | 31.3% (20.6%-42.1%) |
| **Cote D’Ivoire** |  |  |  |  |
| Non-pregnant/breastfeeding women | 10 | 23.0% (23.0%-23.0%) | 14 | 36.8% (36.8%-36.8%) |
| Pregnant/breastfeeding women | 1 | 6.4% (6.4%-6.4%) | 3 | 31.0% (31.0%-31.0%) |
| Women of reproductive age | 11 | 20.4% (20.4%-20.4%) | 17 | 35.9% (35.9%-35.9%) |
| **Eswatini** |  |  |  |  |
| Non-pregnant/breastfeeding women | 50 | 7.2% (5.2%-9.2%) | 102 | 14.6% (11.7%-17.6%) |
| Pregnant/breastfeeding women | 3 | 2.7% (0%-6.0%) | 21 | 21.9% (13.1%-30.7%) |
| Women of reproductive age | 53 | 6.7% (4.9%-8.5%) | 123 | 15.4% (12.6%-18.3%) |
| **Lesotho** |  |  |  |  |
| Non-pregnant/breastfeeding women | 134 | 16.1% (13.4%-18.8%) | 198 | 22.0% (18.9%-25.2%) |
| Pregnant/breastfeeding women | 24 | 22.3% (13.9%-30.7%) | 22 | 18.7% (10.8%-26.5%) |
| Women of reproductive age | 158 | 16.7% (14.1%-19.3%) | 220 | 21.7% (18.6%-24.8%) |
| **Malawi** |  |  |  |  |
| Non-pregnant/breastfeeding women | 84 | 12.2% (8.8%-15.6%) | 142 | 25.6% (21.6%-29.6%) |
| Pregnant/breastfeeding women | 30 | 15.3% (6.9%-23.7%) | 46 | 25.8% (15.9%-35.6%) |
| Women of reproductive age | 114 | 12.9% (9.3%-16.5%) | 189 | 25.7% (21.9%-29.6%) |
| **Namibia** |  |  |  |  |
| Non-pregnant/breastfeeding women | 30 | 4.3% (2.3%-6.3%) | 122 | 19.6% (15.9%-23.3%) |
| Pregnant/breastfeeding women | 5 | 5.4% (0%-11.4%) | 14 | 13.9% (6.4%-21.4%) |
| Women of reproductive age | 35 | 4.4% (2.5%-6.3%) | 136 | 18.9% (15.6%-22.3%) |
| **Uganda** |  |  |  |  |
| Non-pregnant/breastfeeding women | 6 | 40.6% (12.7%-68.4%) | 7 | 33.5% (10.0%-57.1%) |
| Pregnant/breastfeeding women | 2 | 13.7% (0%-32.0%) | 3 | 19.2% (0%-40.7%) |
| Women of reproductive age | 8 | 32.6% (11.0%-54.2%) | 10 | 29.3% (11.2%-47.3%) |
| **Zambia** |  |  |  |  |
| Non-pregnant/breastfeeding women | 79 | 17.0% (12.6%-21.4%) | 77 | 14.5% (11.1%-17.9%) |
| Pregnant/breastfeeding women | 15 | 12.5% (5.7%-19.4%) | 21 | 21.4% (12.3%-30.6%) |
| Women of reproductive age | 94 | 16.1% (12.3%-20.0%) | 98 | 15.8% (12.5%-19.1%) |
| **Zimbabwe** |  |  |  |  |
| Non-pregnant/breastfeeding women | 83 | 12.9% (9.6%-16.2%) | 84 | 11.8% (9.0%-14.6%) |
| Pregnant/breastfeeding women | 17 | 11.2% (4.7%-17.7%) | 15 | 10.4% (3.4%-17.4%) |
| Women of reproductive age | 101 | 12.7% (9.8%-15.6%) | 100 | 11.6% (8.9%-14.3%) |

ART=antiretroviral therapy. CI=confidence interval.

**Table S5. Multivariable model results for adjusted association of sexual violence with suboptimal ART adherence among women of reproductive age living with HIV and on ART ^†^, adjusting for different sets of potential confounders**

|  |  | **aOR**  **(95% CI)** | **P-value^‡^** | **Number of women included in model** |
| --- | --- | --- | --- | --- |
| **Lifetime sexual violence** | No | Yes |  |  |
| **Variables**  **adjusted for** |  |  |  |  |
| Age, region, education | 1 | 1.69 (1.25-2.28) | 0.001 | 5034 |
| Age, region, education, employment | 1 | 1.67 (1.23-2.26) | 0.001 | 5031 |
| Age, region, education, wealth quintile | 1 | 1.66 (1.23-2.24) | 0.001 | 5029 |
| Age, region, education, urban/rural residence | 1 | 1.68 (1.24-2.28) | 0.001 | 5034 |
| Age, region, education, marital status | 1 | 1.70 (1.26-2.30) | <0.001 | 5017 |

ART=antiretroviral therapy. aOR=adjusted odds ratio. CI=confidence interval. HIV=human immunodeficiency virus.

^†^Outcome was ≥1 missed days of ART in the past 30 days.

^‡^Wald test p-value

**Table S6. Multivariable model results for association between sexual violence and ART adherence by pregnant/breastfeeding status among women living with HIV and on ART, by whether women were pregnant/breastfeeding or not pregnant/breastfeeding^†^, adjusting for different sets of potential confounders**

|  | **Pregnant/breastfeeding**  **aOR**  **(95% CI)** | | **P-value^‡^** | **Not pregnant/breastfeeding**  **aOR**  **(95% CI)** | | **P-value^‡^** | **Number of women included in model** | **P-value for interaction by pregnancy/breastfeeding status** |
| --- | --- | --- | --- | --- | --- | --- | --- | --- |
| **Lifetime sexual violence** | No | Yes |  | No | Yes |  |  |  |
| **Variables**  **adjusted for** |  |  |  |  |  |  |  |  |
| Age, region, education | 1 | 4.11 (2.13-7.92) | <0.001 | 1 | 1.39 (1.00-1.93) | 0.05 | 5028 | 0.004 |
| Age, region, education, employment | 1 | 4.07 (2.11-7.87) | <0.001 | 1 | 1.38 (0.99-1.92) | 0.06 | 5025 | 0.004 |
| Age, region, education, wealth quintile | 1 | 4.10 (2.17-7.76) | <0.001 | 1 | 1.37 (0.99-1.90) | 0.06 | 5023 | 0.003 |
| Age, region, education, urban/rural residence | 1 | 4.11 (2.14-7.90) | <0.001 | 1 | 1.38 (1.00-1.92) | 0.05 | 5028 | 0.003 |
| Age, region, education, marital status | 1 | 3.94 (2.04-7.58) | <0.001 | 1 | 1.42 (1.03-1.97) | 0.04 | 5011 | 0.006 |

ART=antiretroviral therapy. aOR=adjusted odds ratio. CI=confidence interval. HIV=human immunodeficiency virus.

^†^Outcome was ≥1 missed days of ART in the past 30 days.

^‡^Wald test p-value

**Table S7. Adjusted association of sexual violence (pressured/attempted forced/physically forced sex) with suboptimal ART adherence among women of reproductive age living with HIV and on ART^†^**

|  | **Number with suboptimal ART adherence / N (%)** | **Crude OR**  **(95% CI)** | **P-value^‡^** | **aOR**  **(95% CI)^§^** | **P-value^‡^** |
| --- | --- | --- | --- | --- | --- |
| **Lifetime sexual violence** |  |  |  |  |  |
| No | 776/4479 (18.5%) | 1 | 0.001 | 1 | 0.002 |
| Yes**^†^** | 163/559 (28.0%) | 1.72 (1.24-2.38) |  | 1.62 (1.19-2.22) |  |

ART=antiretroviral therapy. aOR=adjusted odds ratio. CI=confidence interval. HIV=human immunodeficiency virus. OR=odds ratio.

^†^Outcome was ≥1 missed days of ART in the past 30 days. Sexual violence defined as pressured/attempted forced/physically forced sex. Final adjusted model included n=5034 participants.

^‡^Wald test p-value

^§^Model adjusted for age, region, and education

**Table S8. Adjusted association of sexual violence with suboptimal ART adherence (≥2 missed days of ART in the past 30 days)** **among women of reproductive age living with HIV and on ART^†^**

|  | **Number with suboptimal ART adherence / N (%)** | **Crude OR**  **(95% CI)** | **P-value^‡^** | **aOR**  **(95% CI)^§^** | **P-value^‡^** |
| --- | --- | --- | --- | --- | --- |
| **Lifetime sexual violence** |  |  |  |  |  |
| No | 386/4433 (8.3%) | 1 | <0.001 | 1 | <0.001 |
| Yes | 100/605 (15.8%) | 2.07 (1.42-3.04) |  | 1.97 (1.37-2.83) |  |

ART=antiretroviral therapy. aOR=adjusted odds ratio. CI=confidence interval. HIV=human immunodeficiency virus.

^†^Final adjusted model included n=5034 participants.

^‡^Wald test p-value

^§^Model adjusted for age, region, and education
